# PCMT1 is a potential target related to tumor progression and immune infiltration in liver cancer

**DOI:** 10.1186/s40001-023-01216-1

**Published:** 2023-08-18

**Authors:** Jiahao Liu, Baiying Liu, Yanan Li, Ze Mi, Hongpei Tan, Pengfei Rong

**Affiliations:** 1 Department of Radiology, Third Xiangya Hospital, Central South University, Changsha, 410000 China; 2 Department of Gastrointestinal Surgery, Third Xiangya Hospital, Central South University, Changsha, China

**Keywords:** PCMT1, Liver hepatocellular carcinoma, PI3K–AKT, Prognosis, Immunity

## Abstract

**Background:**

Liver cancer is a prevalent and deadly form of cancer with high incidence and mortality rates. The PCMT1 protein has been linked to cell anti-apoptosis and tumor metastasis, but its significance in liver hepatocellular carcinoma (LIHC) remains largely unexplored.

**Methods:**

We conducted a pan-cancer analysis to examine the expression differences of PCMT1. Kaplan–Meier curves were employed to assess the prognostic impact of PCMT1 on LIHC patients, and we investigated the association between PCMT1 and clinical features, which we validated using a GEO therapeutic dataset. Gene enrichment analysis helped identify signaling pathways associated with PCMT1 expression. Moreover, we evaluated the relationship between PCMT1 and immune cell infiltration, as well as the differences in gene mutations between high-expression and low-expression groups. In vitro and in vivo experiments were performed to assess the effect of PCMT1 on tumor cell lines and mouse tumor models, and potential pathways were explored through gene sequencing.

**Result:**

PCMT1 is highly expressed in most tumors and exhibits a significant association with prognosis in LIHC patients. Pathway enrichment analysis revealed that PCMT1 is involved in cell cycle regulation, immunity, and other processes. Further immune analysis demonstrated that high expression of PCMT1 could reduce tumor-killing immune cell infiltration. In vitro experiments indicated that PCMT1 knockdown could inhibit cancer cell proliferation and migration while promoting apoptosis. In vivo experiments showed that PCMT1 knockdown significantly reduced tumor growth rate, enhanced CD8+T cell infiltration, and increased caspase-3 expression in the tumor area. Gene sequencing suggested that PCMT1 may function through the PI3K–AKT pathway.

**Conclusion:**

Our findings suggest that PCMT1 acts as a promoter of liver cancer progression and may serve as a novel prognostic indicator and therapeutic target for patients with LIHC.

**Supplementary Information:**

The online version contains supplementary material available at 10.1186/s40001-023-01216-1.

## Introduction

Liver cancer ranks as the second leading cause of cancer-related deaths globally, with a continually increasing incidence rate. Projections indicate that by 2025, over 1 million individuals will be diagnosed with liver cancer [1]. Hepatocellular carcinoma (HCC) represents the primary type of liver cancer. Surgical resection serves as the principal treatment for early-stage liver cancer; however, when lymph node or distant metastasis occurs, the survival rate plummets dramatically [2]. Although immune checkpoint inhibitors demonstrate some efficacy in treating liver cancer patients, the response rate remains unsatisfactory [3]. Consequently, identifying critical targets that influence drug tolerance and distant metastasis in liver cancer holds significant importance.

Protein-l-isoaspartate (d-aspartate) *O*-methyltransferase (PCMT1) functions as a protein repair enzyme. Historically, research has predominantly concentrated on its role in protein repair and metabolism, which can affect neural repair [4]. Recent studies have revealed a substantial function for PCMT1 in tumor development and progression [5]. Additionally, it has been discovered that PCMT1 can restructure myocardin and microtubule cells, thus promoting glioblastoma migration and invasion [6]. Emerging research indicates that PCMT1 expression influences immune infiltration in breast cancer, and related studies have corroborated PCMT1 as a crucial driver of ovarian cancer metastasis [7, 8]. Despite investigations into PCMT1's role in various tumors, its significance in liver cancer remains elusive. Therefore, we utilized publicly available databases and integrated bioinformatics methods to conduct a preliminary investigation into the relationship between PCMT1 expression and liver cancer patient prognosis. Moreover, we explored its potential mechanisms in LIHC through RNA sequencing and pertinent experiments.

## Methods

### Data acquisition

In this study, we downloaded data from the TCGA website for 33 types of cancers, obtaining a total of 11,116 samples, including gene expression information and clinical characteristics of tumor and non-tumor samples. We further analyzed the relevant data specific to liver cancer. The genetic symbols were obtained directly from the database, and gene expression data were normalized using log2(TPM + 0.001). We extracted survival and clinical phenotype data for liver cancer patients and excluded samples without prognostic information, resulting in 374 tumor samples and 50 samples of normal adjacent tissue. Our primary focus is the correlation between PCMT1 expression and prognosis of liver cancer patients. We utilized R software to analyze the relationship between PCMT1 expression and immune cell infiltration levels in liver cancer.

### Functional enrichment analysis of differential expression genes (DEGs)

Utilizing R software, we initially identified differentially expressed genes (DEGs) between high and low PCMT1 expression groups [9]. We used a heatmap to visually analyze the Spearman correlation between the top 50 DEGs and PCMT1, and further conducted functional enrichment analysis, including Gene Ontology (GO), Kyoto Encyclopedia of Genes and Genomes (KEGG), and Gene Set Enrichment Analysis (GSEA). Finally, we performed GSEA analysis based on the "Molecular Signatures Database" c2.cp.kegg.v7.1.symbols to assess possible underlying mechanisms [10]. We selected a random sample size of 100, and only results with *P* < 0.05 were considered to be statistically significant.

### PCMT1 and tumor immunity

We used the ESTIMATE algorithm to analyze the relationship between immune cell infiltration and PCMT1 expression in tumors [11, 12]. Additionally, we used the "corrplot" and "ggpubr" packages in R to analyze the correlation between PCMT1 levels and selected immune-related gene markers. TCIA algorithm was used to predict the impact of PCMT1 expression on the response to immunotherapy in LIHC patients.

### Analysis of TMB (tumor mutational burden) and tumor gene mutations.

We obtained somatic mutation data for all LIHC patients from TCGA, calculated the tumor mutation burden (TMB) score using R software, and analyzed the correlation between TMB and PCMT1 expression levels [11, 13]. In addition, we further analyzed the differences in gene mutations between the high and low PCMT1 expression groups of LIHC samples.

### Drug sensitivity prediction

We employed the "prophetic" R package to compute the IC50 of chemotherapy drugs [14]. This method is primarily utilized to predict the effectiveness of a substance in inhibiting a specific biological or biochemical process. Using this approach, we can assess the potential correlation between PCMT1 expression and drug sensitivity, thereby screening potential candidate drugs for LIHC patients.

### Patients tissue sample acquisition

The tumor tissue and corresponding adjacent tissue of liver cancer patients were obtained from the Third Xiangya Hospital. The tumor tissue was pathologically diagnosed as LIHC.

### Cell culture

We procured hepatic carcinoma cell lines (HepG2 and Hep1-6) from the Shanghai Cell Bank, and we cultured them in DMEM high glucose medium supplemented with 10% fetal bovine serum and 1% penicillin–streptomycin. The cells were maintained in a culture environment with a stable temperature of 37 °C and a CO_2_ concentration of 5%. To obtain LIHC cells with PCMT1 knockdown, shRNA targeting PCMT1 was transfected when the cell density achieved 50–70% by Lipofectamine3000, cells were screened for antibiotics to enrich cells with integrated shRNA structures. The expression level of target genes was evaluated by western blotting to determine the knockout efficiency of stable cell lines.

### Cell counting kit-8 (CCK-8) assay

We seeded normal and PCMT1 knockdown cells in 96-well plates at a density of 2 × 10^3^ cells per well. After adding CCK8 reagent, the cells were incubated for 1 h and the cell proliferation was measured at 450 nm.

### Transwell assay

The corresponding cells were seeded at a density of 2.0 × 10^5^ cells per well and cultured for an additional 48 h. The migration ability of the hepatic carcinoma cells was determined using the Transwell assay.

### Apoptosis assay

The Apoptosis Detection Kit (catalog #556547, BD) was used to investigate the impact of PCMT1 on cell apoptosis. The experimental procedures were performed according to the instructions provided with the kit, and flow cytometry was used to detect the results.

### Establishment of mouse tumor model

The hep1-6 cells are washed with PBS to obtain a suspension of 1 × 10^7^ cells/ml. Adult C57BL/6 male mice are used for the tumor model. Mice are acclimatized for a week before the experiment, and their health is monitored daily. The mice are anesthetized with isoflurane, and the Hep1-6 cell suspension (100 µL) is injected subcutaneously into the right flank of each mouse. The mouse tumor size was measured every 3 days using a vernier caliper. Once the tumor reached an appropriate size, the mouse was euthanized, and the tumor was collected for further analysis, such as histological examination, immunohistochemistry, immunofluorescence and gene expression analysis.

### Detection of immune cell infiltration

The mouse tumor tissue is dissected and mechanically dissociated with scissors and forceps. The tumor tissue is then enzymatically digested using a cocktail of collagenase, hyaluronidase, and DNase. The digested tumor tissue is filtered through a 70-µm cell strainer to obtain a single-cell suspension, stained with corresponding T cell antibodies, and analyzed by flow cytometry. The infiltration and polarization of macrophages in the tumor were evaluated by immunofluorescence staining.

### Statistical method

In our study, we aimed to investigate the association between PCMT1 expression and prognosis in liver cancer patients by analyzing the clinical indicators of OS and PFS. We used the Wilcoxon log-rank test to compare the sum of gene expression *z*-scores between cancerous and adjacent normal tissues, and the Kruskal–Wallis test to assess the differences in PCMT1 expression among different tumor stages. The survival analyses were conducted using KM curves, the log-rank test, and Cox proportional hazards regression models. Additionally, we performed Spearman’s test to evaluate the correlation between PCMT1 expression and clinical parameters. All statistical analyses were carried out using the R programming language (version 4.1.0; R Foundation), and a two-sided *P*-value less than 0.05 was considered statistically significant.

## Results

### PCMT1 expression is increased in liver cancer patients

**Fig. 1:**
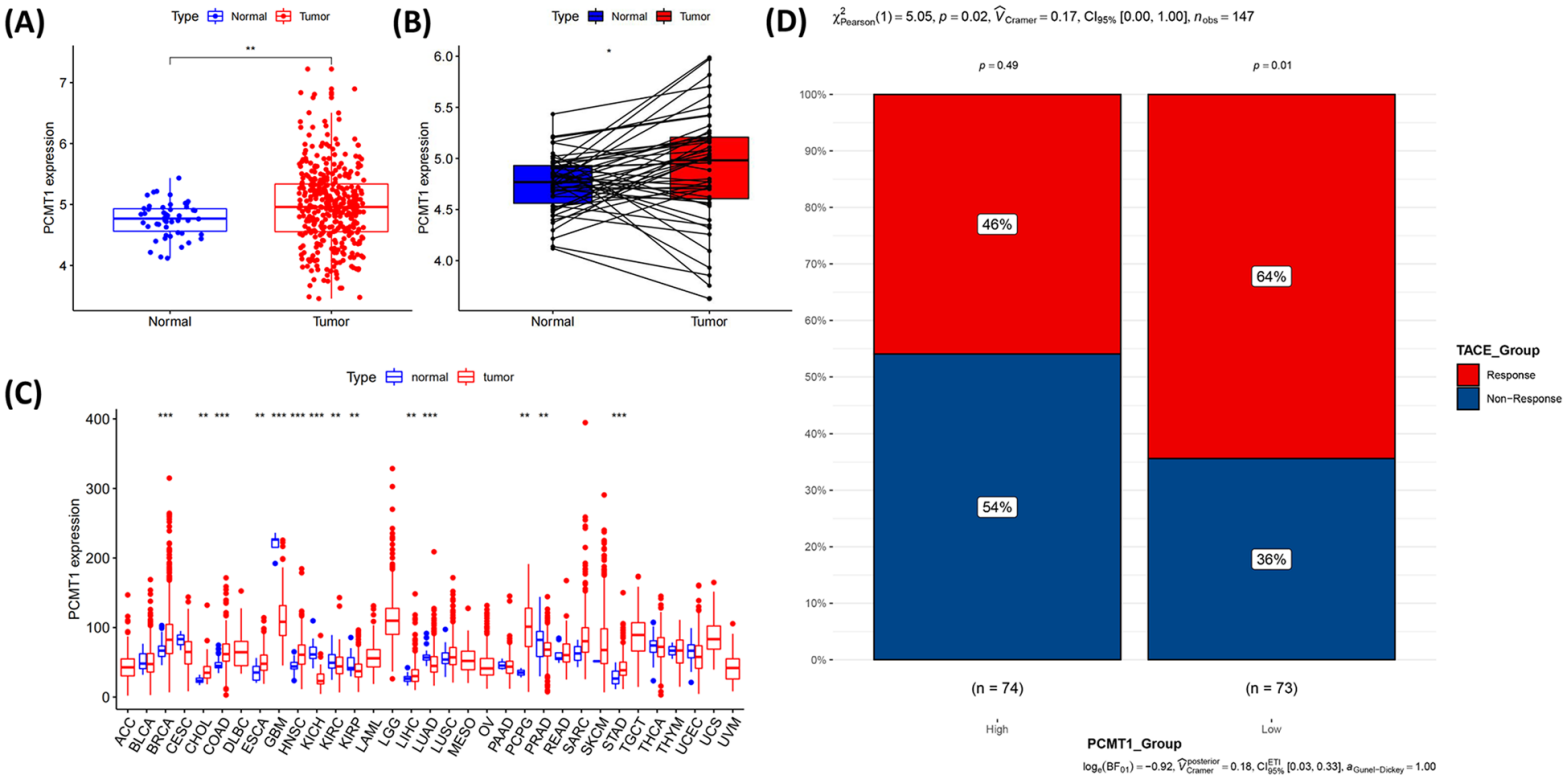
PCMT1 is highly expressed in LIHC and many other tumors. **A** Analysis of PCMT1 expression in liver cancer and adjacent normal tissues in the TCGA database. **B** TCGA database and statistical analyses of PCMT1 expression in 58 pairs of LIHC tissues and adjacent normal tissues. **C** The mRNA level of PCMT1 in TCGA. The blue and red bar graphs indicate normal and tumor tissues, respectively. **D** Relationship between TACE therapy reactivity and PCMT1 expression. *P < 0.05; **P < 0.01; ***P < 0.001; ****P < 0.0001

We analyzed the mRNA expression of PCMT1 in 33 types of cancer, observing that its expression levels varied significantly among different cancers. However, PCMT1 was predominantly upregulated in cancer (Fig. 1A) and PCMT1 expression was associated with higher clinical stage (Additional file 1: Fig. S1). Overall, we discovered that PCMT1 expression was considerably increased in LIHC samples compared to adjacent normal tissues (Fig. 1B). We further analyzed 50 pairs of tumor samples and corresponding adjacent normal tissues from the same patient, finding that PCMT1 expression was significantly elevated in tumor tissues (Fig. 1C). To validate the role of PCMT1 in LIHC treatment, we selected a dataset of LIHC patients who underwent transarterial chemoembolization (TACE) treatment from the Gene Expression Omnibus (GEO) (GSE104580). We first divided HCC patients into two groups according to the level of PCMT1 expression, and our analysis showed that patients with low PCMT1 expression accounted for more of TACE-responsive patients. (Fig. 1D). These results suggest that PCMT1 may play an important role in the progression of liver cancer.

### PCMT1 expression was associated with the poor prognosis and clinicopathological factors of LIHC

**Fig. 2:**
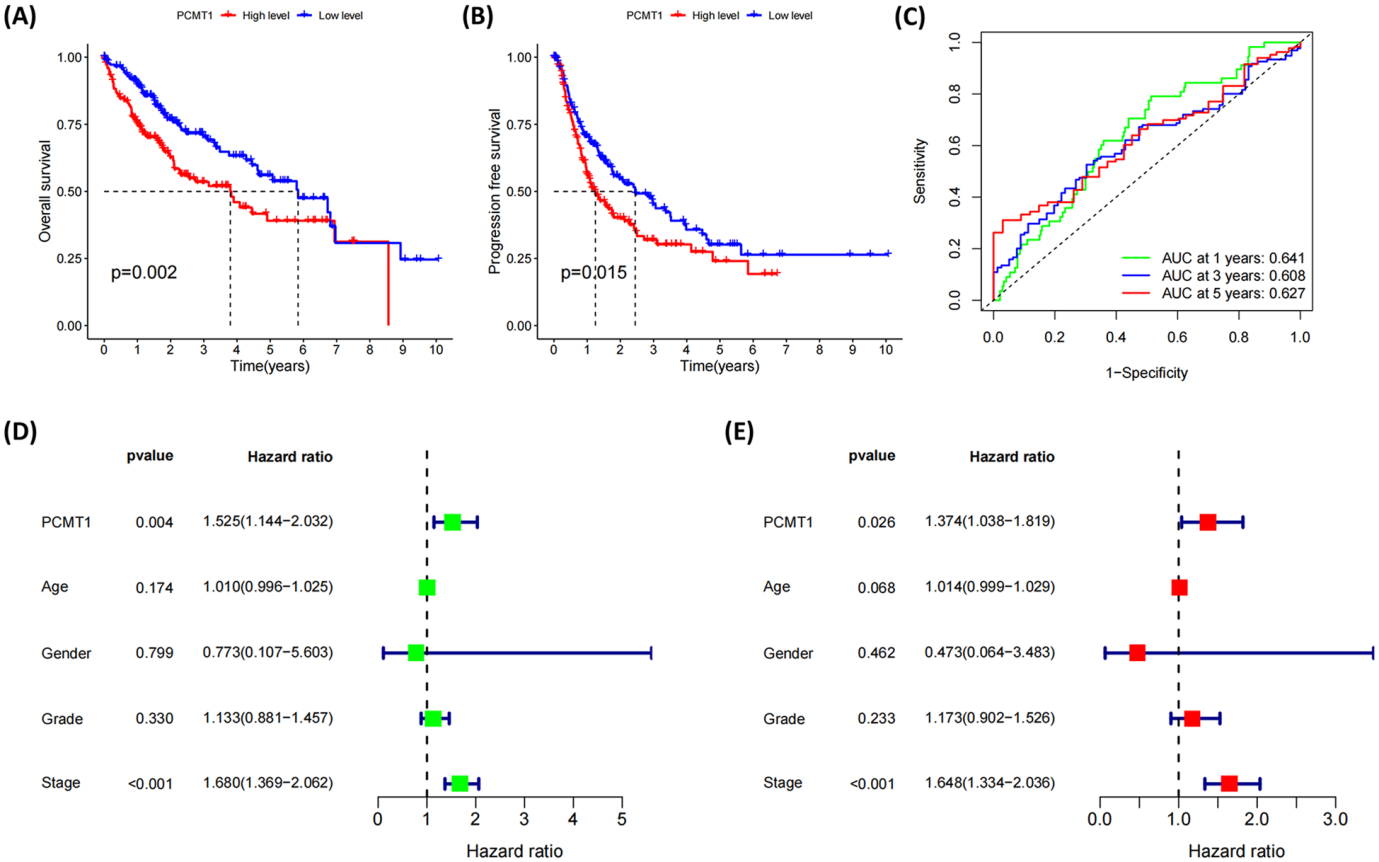
PCMT1 is associated with survival in patients with liver cancer. **A** The relationship between PCMT1 and OS in liver cancer patients; **B** the relationship between PCMT1 and PFS in liver cancer patients; **C** ROC curve of prognostic prediction of liver cancer patients, univariate (**D**) and multivariate survival analysis (**E**) combined with clinical information of PCMT1

Our results showed that PCMT1 was significantly upregulated in T3 and T4 stages compared to T1 stage and in stage III compared with stage I (Additional file 1: Fig. S1). It is noteworthy that higher expression of PCMT1 in liver cancer patients is associated with poorer OS and PFS (Fig. 2A, B). We further plotted ROC curves to evaluate the prognostic value of PCMT1, which showed that the AUC values corresponding to 1-year, 3-year, and 5-year survival were 0.641, 0.608, and 0.627, respectively (Fig. 2C). Furthermore, univariate Cox regression analysis identified stage and PCMT1 as prognostic factors for LIHC patients (Fig. 2D), and multivariable Cox regression analysis revealed that both high expression of PCMT1 and advanced tumor stage were independent unfavorable prognostic factors for LIHC patients’ prognosis (Fig. 2E). Our research findings indicate a close correlation between PCMT1 expression and clinical features as well as prognosis of LIHC patients, highlighting its potential as a therapeutic target and prognostic assessment in liver cancer.

### Differential gene pathway analysis associated with PCMT1

**Fig. 3:**
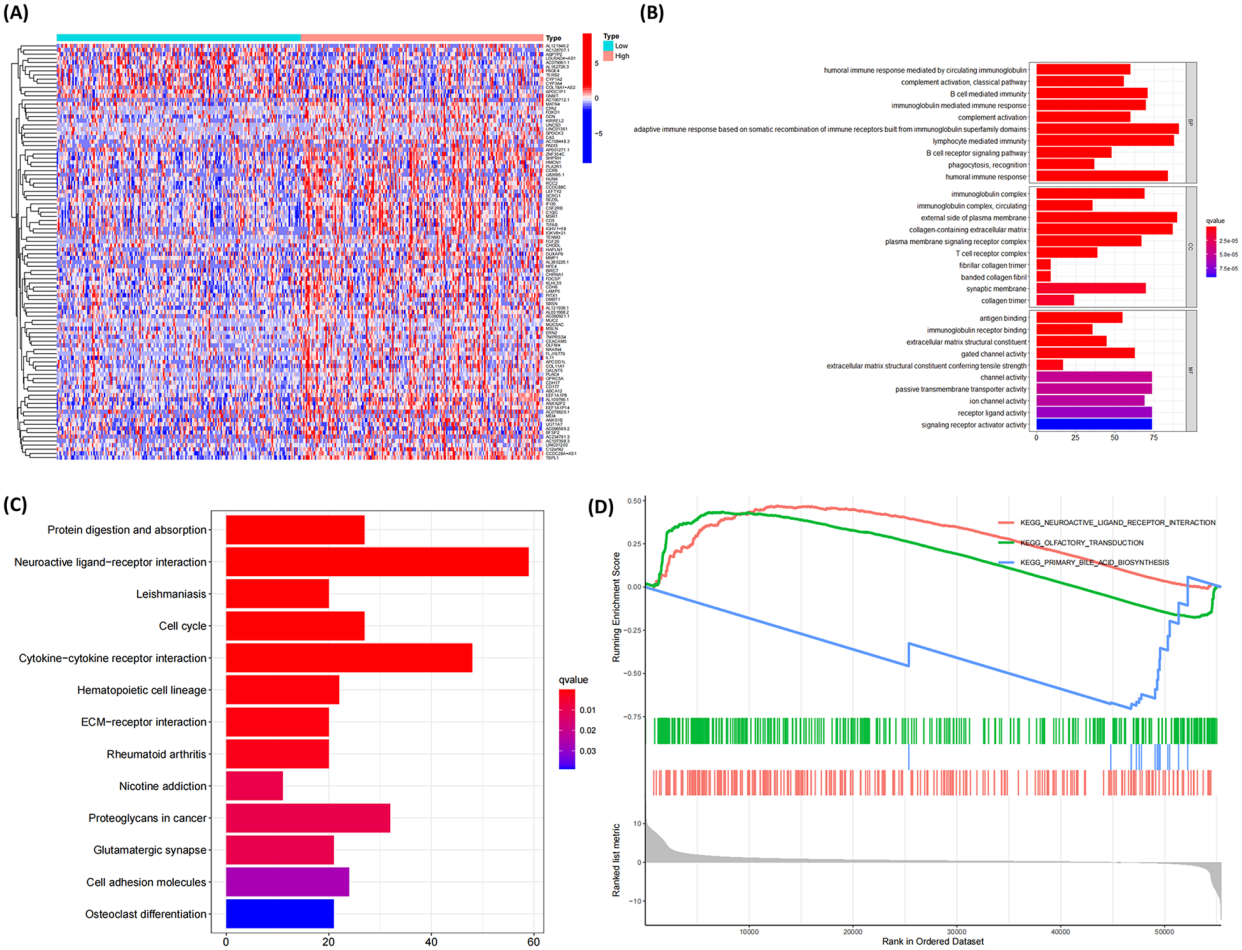
Differential gene analysis of tumor samples with high and low PCMT1 expression. **A** Heat map of the top 50 differentially expressed genes of PCMT1. **B** GO analysis of differentially expressed genes. **C** KEGG analysis of differentially expressed genes. **D** GSEA analysis of differentially expressed genes

Firstly, we identified differentially expressed genes (DEGs) based on the expression levels of PCMT1, with a total of 2582 genes exhibiting differential expression. The heatmap in Fig. 3A illustrates the relationship between the top 50 DEGs and the expression levels of PCMT1. Furthermore, Gene Ontology (GO) term pathway enrichment analysis, as shown in Fig. 3B, revealed that PCMT1 affects immune functions such as humoral immune response mediated by circulating immunoglobulin, B cell-mediated immunity, and T cell receptor complex. KEGG pathway analysis results demonstrated that DEGs were primarily enriched in neuroactive ligand–receptor interaction, cytokine–cytokine receptor interaction, proteoglycans in cancer, cell cycle, and cell adhesion molecules (Fig. 3C). Subsequently, we performed Gene Set Enrichment Analysis (GSEA) using the KEGG pathway genome. As shown in Fig. 3D, the expression levels of PCMT1 were associated with the neuroactive ligand–receptor pathway, olfactory transduction, and primary bile acid biosynthesis.

### Immune infiltration analysis of PCMT1 in LIHC cancer

**Fig. 4:**
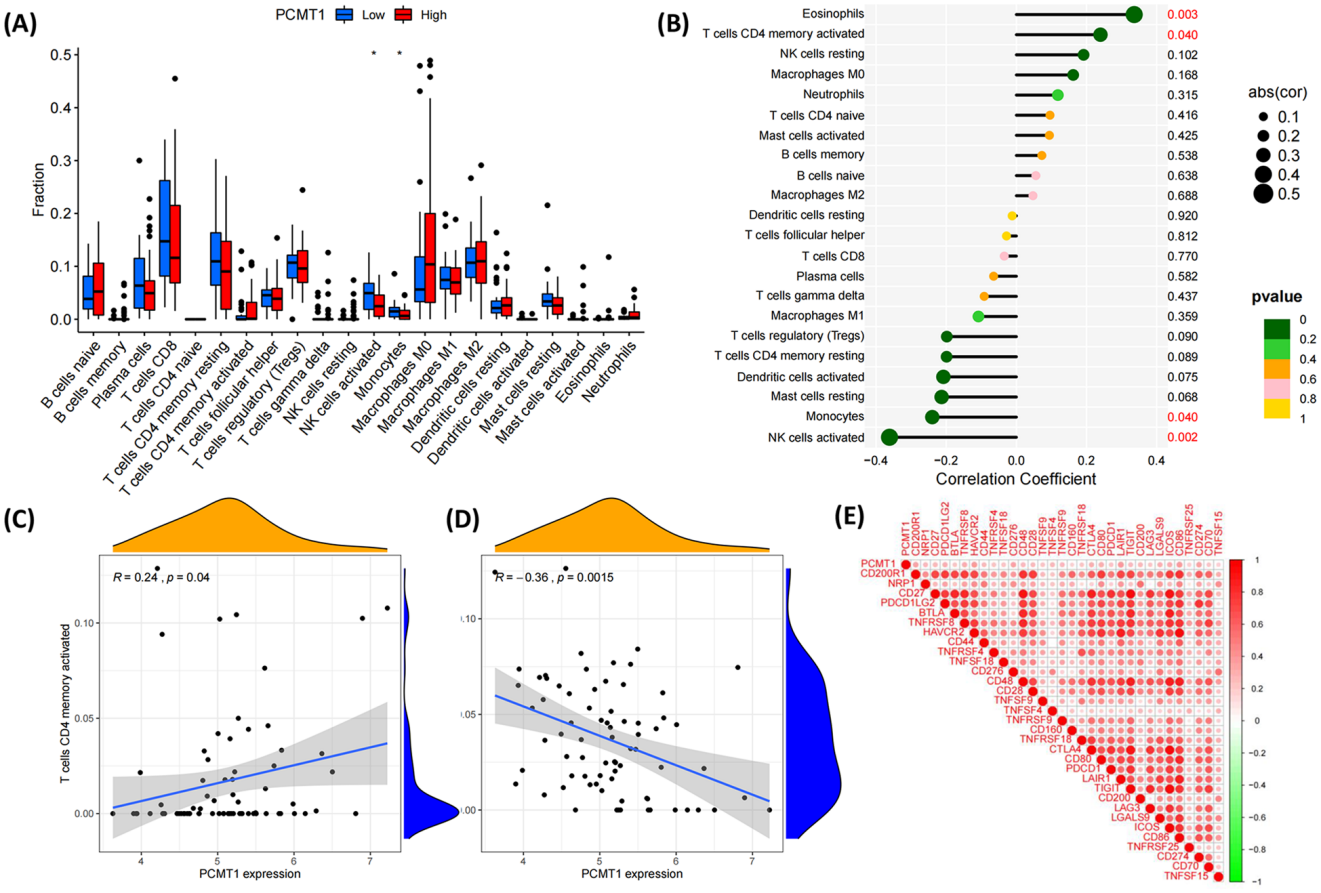
The expression of PCMT1 is associated with immune infiltration. **A** The relationship between PCMT1 and immune cell infiltration; **B** the correlation parameters between PCMT1 and various immune cell infiltration; **C** the correlation between PCMT1 and CD4memoryT cells; **D** the correlation between PCMT1 and NK cells; **E** the correlation heat map between PCMT1 and immune checkpoint

The immune microenvironment plays a critical role in tumor progression and treatment. We assessed the impact of differential PCMT1 expression on immune cell infiltration. The results showed that PCMT1 significantly affects immune cell infiltration in tumors (Fig. 4A). Using the ssGSEA algorithm, we further evaluated the correlation between PCMT1 expression and immune cell infiltration, observing a negative correlation between PCMT1 and eosinophil and activated NK cell infiltration, but a significant positive correlation with monocyte and CD4+T cell infiltration (*p* < 0.05, Fig. 4B–D). The expression of immune checkpoints affects the composition of the entire immune microenvironment; therefore, we further evaluated the relationship between PCMT1 and 33 immune checkpoints in LIHC. The results showed that the PCMT1 level is positively correlated with the levels of various immune checkpoints in LIHC, especially HAVCR2, LAIR1, CD86, and CD274 (Fig. 4E).

### PCMT1 was associated with cancer-related gene mutation and drug sensitivity

**Fig. 5:**
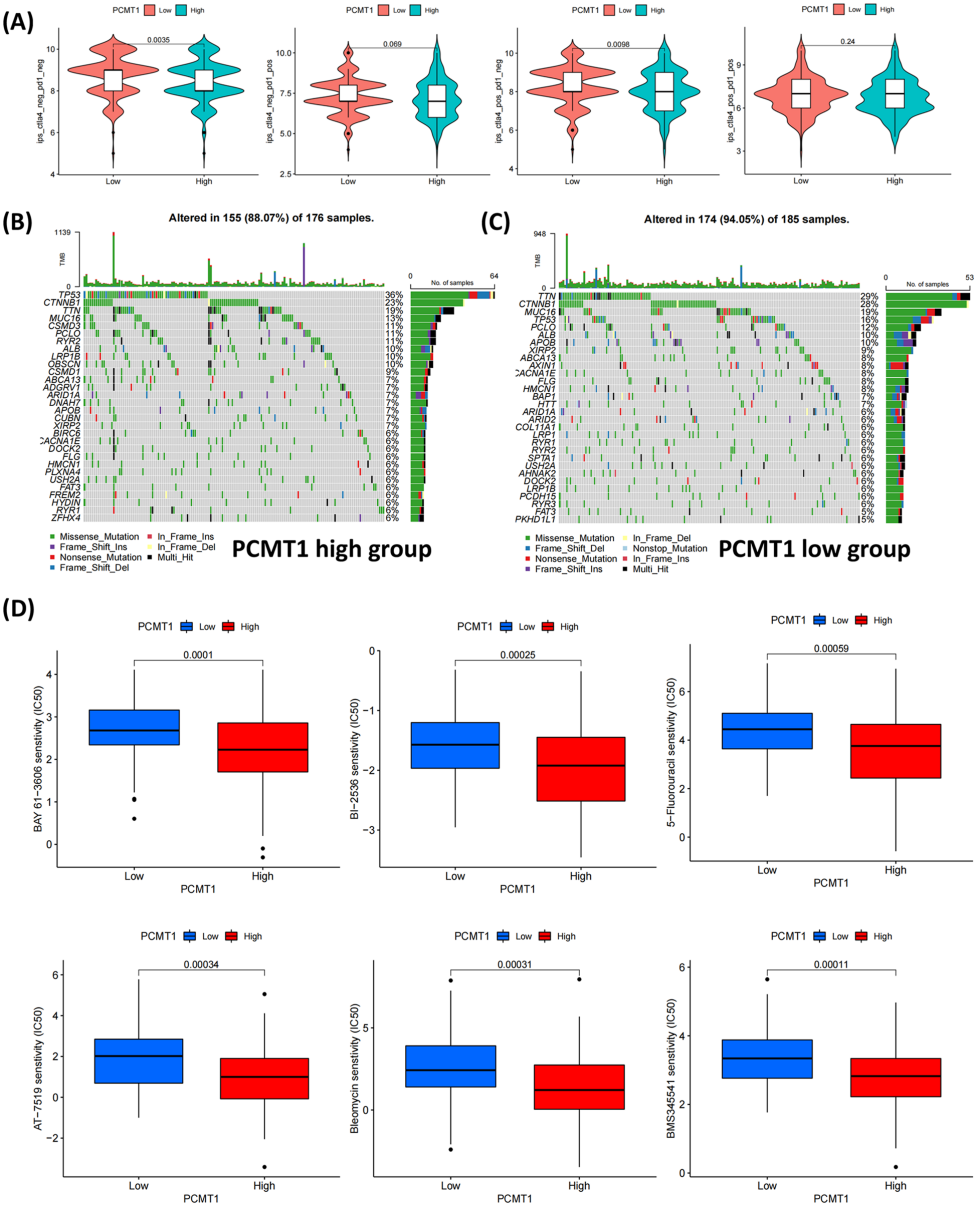
PCMT1 affects TP53 mutations and immunotherapy outcomes. **A** The correlation between PCMT1 expression and ICI treatment was predicted by IPS score, a lower score predicted a lower immunotherapy response rate. **B** The top30 mutant genes with high expression of PCMT1 were grouped. **C** The top30 mutant genes with low expression of PCMT1 were grouped. **D** Analysis of drug sensitive screening for PCMT1

To explore the expression of PCMT1 and its responsiveness to immune therapy, we employed immune phenotyping (IPS) to evaluate the response to immune checkpoint inhibitors (ICI) treatment. According to the expression of CTLA4 and PD1, the patients were divided into four groups to evaluate the influence of the expression level of PCMT1 on the sensitivity to ICI treatment. Our results showed that high expression of PCMT1 in CTLA4 and PD1 double negative group and in CTLA4 positive and PD1 negative group would reduce the sensitivity to ICI treatment (Fig. 5A). To determine the differences in cancer-associated gene mutations between the PCMT1 high and low-expression groups, we assessed the mutation status of representative genes in each group. PCMT1 expression was not related to tumor mutation burden (Additional file 2: Fig. S2). The general information on representative gene mutations in each group is presented (Fig. 5B, C). From the results, TP53, CTNNB1, TTN, MUC16, and CSMD3 were the top five genes with the highest mutation frequency in the high-expression group, while TTN, CTNNB1, MUC16, TP53, and PCLO were the top five genes with the highest mutation frequency in the low-expression group. Therefore, PCMT1 may affect the mutation status of cancer-related genes.

To further investigate drugs that may be effective against high expression of PCMT1, we compared the estimated IC50 levels of 138 chemotherapy drugs or inhibitors. Representative drugs are shown in Fig. 5D. Lower values may indicate higher sensitivity to the drug. We found that BAY 61-3606, BI-2536, 5-fluorouracil, AT-7519, bleomycin, and BMS345541 are potential candidate drugs for treating patients with PCMT1 high expression.

### Knockdown of PCMT1 inhibited the growth, migration and increased apoptosis of liver cancer cells

**Fig. 6:**
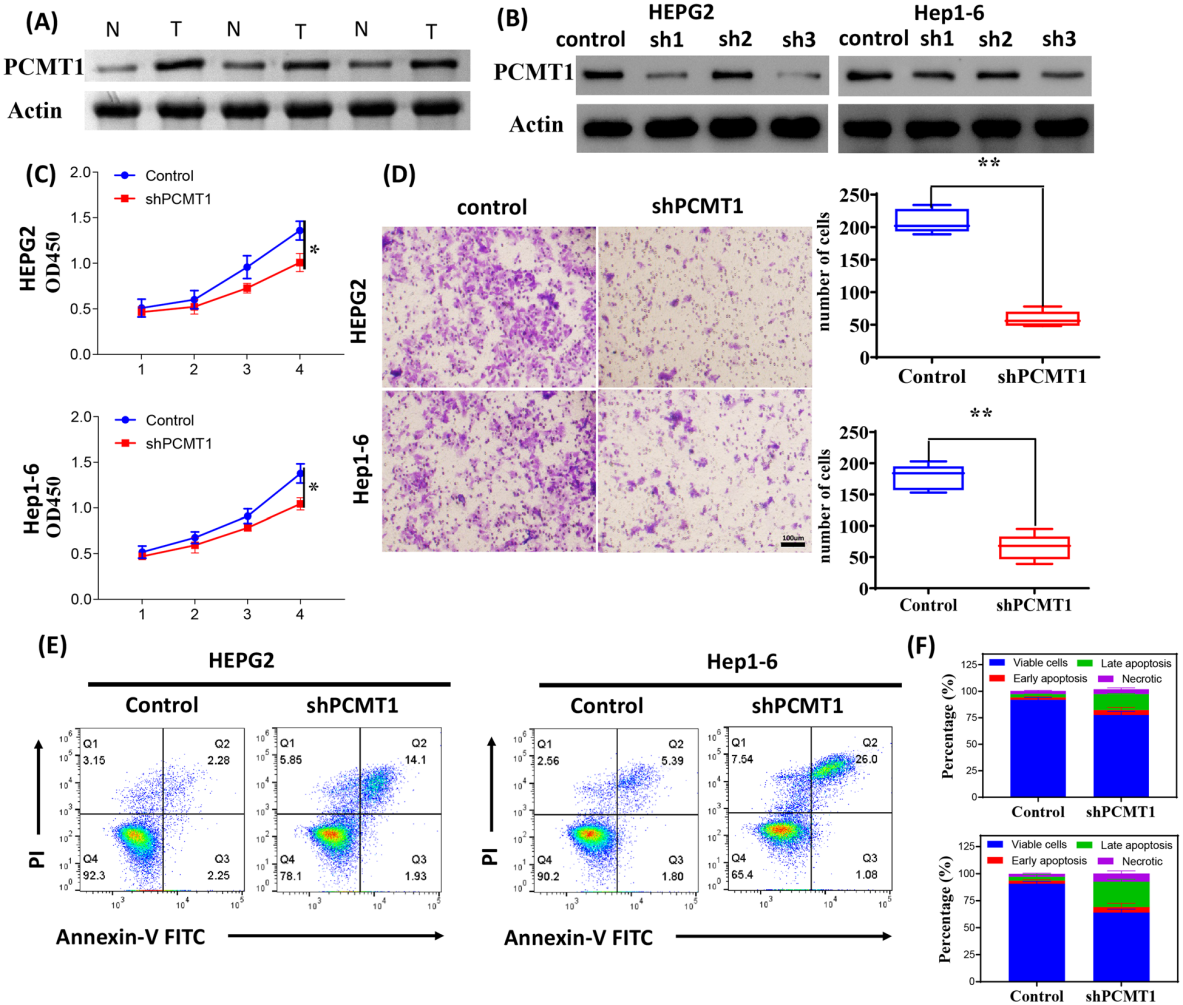
PCMT1 affects the growth and invasion of hepatocellular carcinoma cells. **A** The expression of PCMT1 in tumor tissue and adjacent to tumors in patients with liver cancer. **B** The expression of PCMT1 knocked down in two tumor cells. **C** The effect of PCMT1 knocked down on the growth of tumor cells. **D** The migration experiment of two tumor cells after PCMT1 knocked down. **E, F** The flow cytometry analysis of the apoptosis of two liver cancer cells after PCMT1 knocked down and corresponding statistical charts

Initially, we conducted Western blotting to examine the protein expression levels of PCMT1 in the tumor tissue and adjacent non-tumor tissue of liver cancer patients. The results revealed a significant increase in PCMT1 expression in the tumor region (Fig. 6A).

In vitro experiments demonstrated that the knockdown of PCMT1 significantly inhibited the proliferation of HEPG2 and Hep1-6 cells (Fig. 6B, C). Additionally, the Transwell assay confirmed that the migration ability of the cells was significantly reduced following PCMT1 knockdown (Fig. 6D). Furthermore, flow cytometry analysis revealed a significant increase in cell apoptosis after PCMT1 knockdown (Fig. 6E, F). These findings indicate that PCMT1 may play a vital regulatory role in liver cancer.

### RNA sequencing showed that PCMT1 was associated with PI3K pathway and apoptosis

**Fig. 7:**
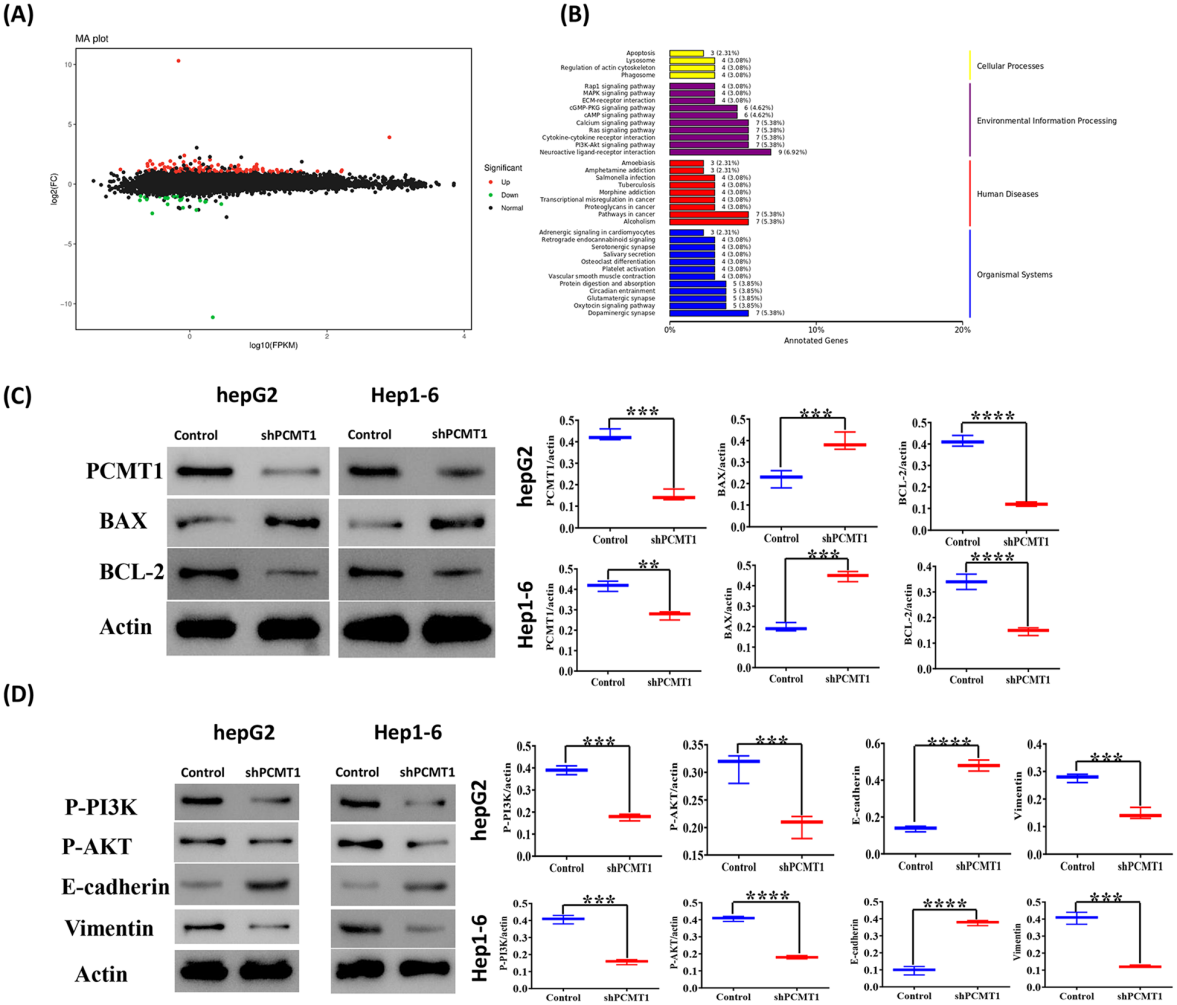
PCMT1 affects the PI3K–AKT pathway. **A** Volcano map of differentially expressed genes obtained by RNA sequencing after PCMT1 knockdown. **B** KEGG analysis of differentially expressed genes. **C** The effect of PCMT1 knockdown on the levels of apoptosis-related proteins and quantitative analysis of protein levels. **D** The effect of PCMT1 knockdown on the phosphorylation of PI3K, AKT, the expression of EMT-related proteins and quantitative analysis of protein levels

We employed RNA transcriptome sequencing to explore the potential mechanism of PCMT1's role in liver cancer. The results showed that the knockdown of PCMT1 led to the upregulation of 112 genes and the downregulation of 22 genes, Go analysis showed that differentially expressed genes were mainly related to intrinsic component of membrane (Fig. 7A). KEGG analysis of the differentially expressed genes indicated that PCMT1 may affect apoptosis and the PI3K–AKT pathway (Fig. 7B). We validated the expression of apoptosis-related proteins, BAX and BCL-2, following PCMT1 knockdown in two liver cancer cell lines. The results demonstrated a significant increase in the expression of BAX after PCMT1 knockdown, the expression of BCL-2 protein was significantly decreased (Fig. 7C). Additionally, we confirmed that the phosphorylation levels of PI3K and AKT were reduced following PCMT1 knockdown. Lastly, we examined EMT-related proteins and found that PCMT1 knockdown could decrease EMT in liver cancer cells (Fig. 7D).

### Knockdown of PCMT1 inhibited tumor growth and promoted tumor apoptosis

**Fig. 8:**
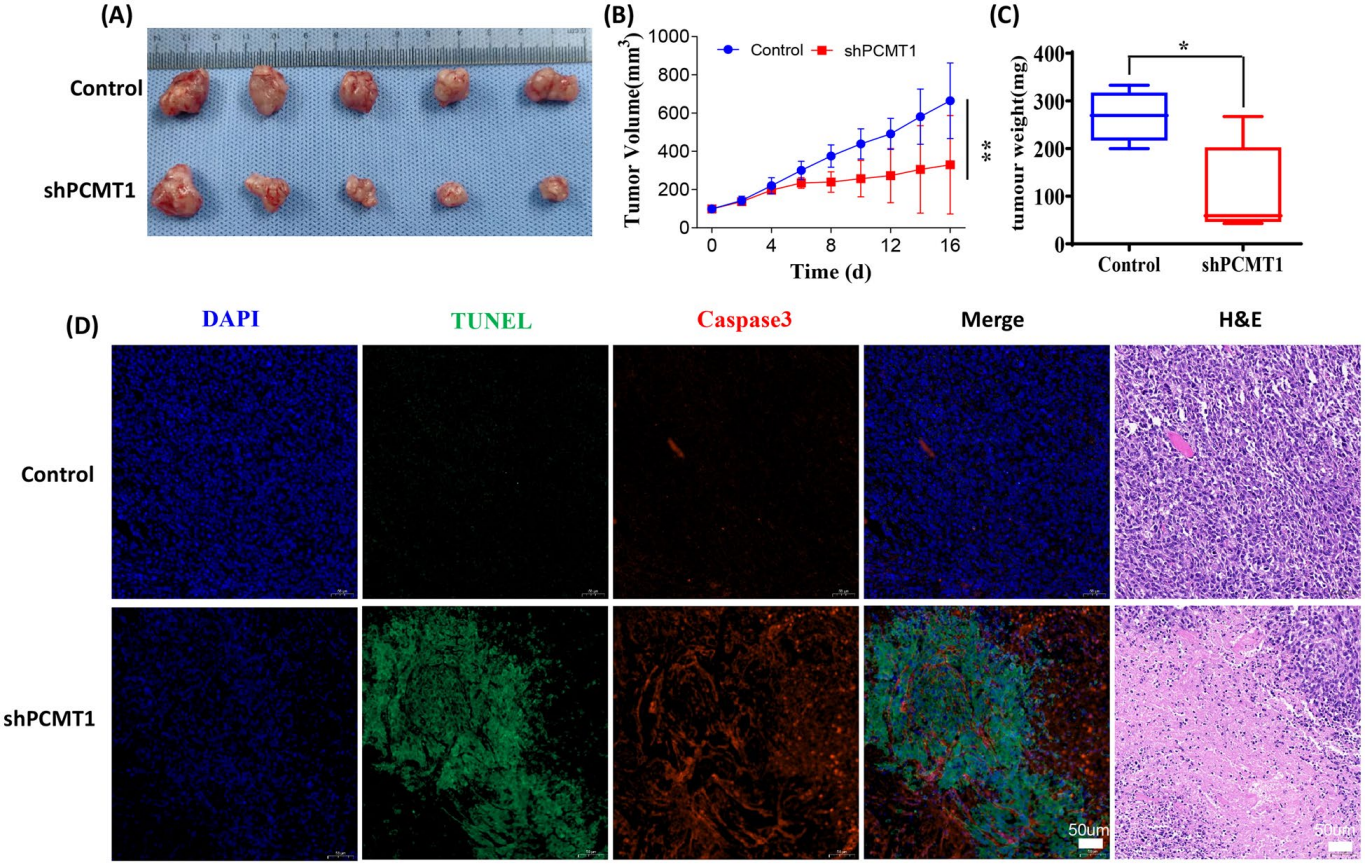
PCMT1 affects tumor growth and apoptosis. **A** Tumor taken from mice in groups with or without PCMT1 knockdown. **B** The tumor volume from mice in two groups over time; **C** comparison of tumor weight between two groups with or without PCMT1 knockdown; **D** fluorescence staining of TUNEL and caspase-3 and corresponding HE staining in tumor samples of mice in two groups

We further studied the effect of PCMT1 on tumor growth in vivo, and the results showed that the tumor growth curve was significantly slower and the tumor weight was significantly lower in the mice with PCMT1 knockdown (Fig. 8A–C). After further staining of tumor tissues, it was found that knockdown PCMT1 could significantly increase the apoptosis of tumor region. This was further confirmed by caspase-3 staining, and HE staining was also performed, which showed that apoptosis of the tumor area was very obvious after knockdown of PCMT1 (Fig. 8D).

### PCMT1 affects the infiltration and function of immune cells in tumor area

**Fig. 9:**
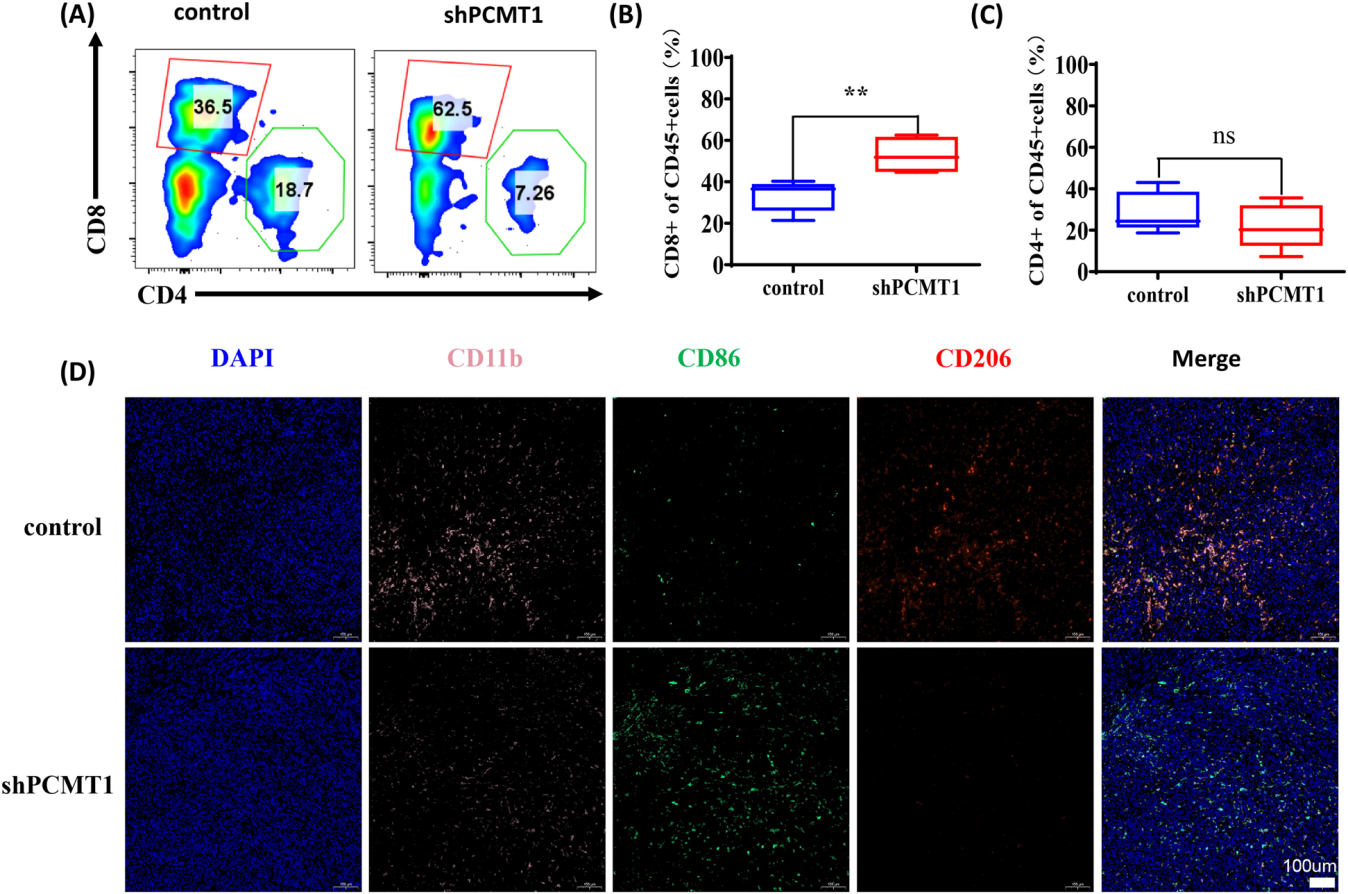
PCMT1 affects immune cell infiltration and macrophage phenotype in the tumor area. **A** The effect of PCMT1 knockdown on the infiltration of CD4+T cells and CD8+T cells in tumor area; **B** statistical analysis of CD8+T cell infiltration in tumor area under different treatments; **C** statistical analysis of CD8+T cell infiltration in tumor area under different treatments; **D** fluorescence staining of CD11b and macrophage subtype markers CD86 and CD206 in two groups of tumors

Bioinformatics immunoassay results showed that PCMT1 could affect immune infiltration in the tumor region, so we further examined the immune cell infiltration in the tumor region of mice in the two groups with or without PCMT1 knockdown. We detected the infiltration of CD4 and CD8T cells in the tumor area by flow cytometry, and the results showed that the infiltration of CD8+T cells was significantly increased after PCMT1 knockdown (Fig. 9A–C). The immunofluorescence staining of tumor sections showed that the expression of CD86 was significantly increased after PCMT1 knockdown, while the expression of CD206 was decreased (Fig. 9D). This may be related to the promotion of M1 polarization of macrophages, thus promoting anti-tumor immunity, which may also be the possible reason for the slower tumor growth after PCMT1 knockdown.

## Discussion

In this study, we discovered that PCMT1 was overexpressed in hepatocellular carcinoma. Additionally, we investigated the relationship between PCMT1 mRNA expression and various clinical variables such as tumor status, lymph node status, distant metastasis, and pathological staging. The results indicated that high PCMT1 expression was associated with poor prognosis and was an independent prognostic factor for survival. Through gene expression profiling analysis and in vitro and in vivo experiments, we further explored the potential mechanisms of PCMT1 in the progression of liver cancer.

We first analyzed the differentially expressed genes between PCMT1 high and low expression patients. KEGG and GO analysis revealed that these genes were mainly enriched in immune-related pathways and protein digestion and absorption, and could also affect cell cycle. Further GSEA analysis revealed that PCMT1 affects the synthesis of primary bile acids. Previous studies have demonstrated that primary bile acids can recruit NKT cells to suppress liver cancer [15]. Based on our analysis, PCMT1 is likely to impact the proliferation of liver cancer cells as well as the formation of the tumor microenvironment. We then analyzed the differences in immune microenvironment between the two groups. Our results showed that there were significant differences in the proportion of immune cells between PCMT1 high and low expression groups, and the infiltration level of different cells was significantly correlated with PCMT1 copy number. CD4+memory T cells infiltrated more in PCMT1 high expression patients while NK cells infiltrated less. NK cells and CD4+T cells have been proven to play important roles in liver cancer and are associated with various treatment outcomes [16–20]. Therefore, PCMT1 expression is likely to affect the tumor microenvironment. We further analyzed and found that PCMT1 expression was positively correlated with immune checkpoint expression, which could be one of the reasons for the poor survival rate in PCMT1 high expression patients. PCMT1 expression was not related to tumor mutation burden, but immunotherapy data analysis showed that the immunotherapy effective rate might be lower in PCMT1 high expression patients. Therefore, based on our results, PCMT1 is likely to inhibit tumor immunity and promote tumor progression. In addition to immunotherapy, TACE therapy is also an important treatment for liver cancer [21–23]. Our analysis of the database results revealed that the expression of PCMT1 was significantly lower in TACE-responsive patients compared to TACE non-responsive patients. Therefore, PCMT1 can also serve as a target for predicting the therapeutic effect and can aid in providing more accurate treatment for patients.

In order to further investigate the role of PCMT1 in liver cancer, we first analyzed the expression of PCMT1 in tumor tissue and adjacent tissue of liver cancer patients. The results showed that PCMT1 expression was significantly higher in liver cancer tissue than in adjacent tissue. Then, we established a stable cell line with low expression of PCMT1 through shRNA, in order to further study the role of PCMT1 in liver cancer cell lines. Through CCK8 and Transwell experiments, we found that knocking down PCMT1 could significantly inhibit the growth and migration of liver cancer cells. Further flow cytometry results showed a significant increase in apoptosis of cells after PCMT1 knockdown. PCMT1 may affect cell apoptosis through various mechanisms. Some studies suggest that PCMT1 can prevent cell apoptosis by affecting intracellular ROS levels and caspase-3/9 activity [24]. Additionally, PCMT1 can also inhibit cell apoptosis induced by overexpression of Bax [25]. Furthermore, after subarachnoid hemorrhage, MST1 is activated and promotes neuronal apoptosis, which can be significantly inhibited by PCMT1 [26]. Moreover, there is evidence that downregulation of PCMT1 expression after DNA damage induced by various factors can significantly increase cell apoptosis [27]. The mechanism of radiotherapy and some anti-tumor drugs involves the activation of reactive oxygen species or DNA damage [28], thus PCMT1 is likely to be associated with radiotherapy resistance and drug resistance.

To explore the possible mechanisms of PCMT1 in liver cancer progression, we conducted RNA transcriptome sequencing and analyzed the differentially expressed genes between the two groups with or without PCMT1 knockdown. The results showed that PCMT1 may affect cell apoptosis and the PI3K–Akt pathway. The PI3K–Akt pathway is widely studied in tumors, and its activation can promote tumor proliferation, invasion, and metastasis, and is also related to tumor treatment resistance [29–32]. To validate the sequencing results, we conducted further experiments and found that PCMT1 knockdown can promote the high expression of apoptosis-related proteins, and reduce the phosphorylation of PI3K and Akt, as well as inhibit EMT. Therefore, our results suggest that PCMT1 may be a crucial molecule in liver cancer progression.

Many studies have demonstrated that tumor cells can influence tumor progression by influencing tumor immune microenvironment [33, 34]. In order to investigate the effects of PCMT1 on tumor growth and the tumor immune microenvironment, we further studied the role of PCMT1 in vivo by establishing a mouse model of liver cancer, we found that the tumor volume grew more slowly, and the apoptotic staining and caspase-3 expression were significantly higher in the tumor area in the knockdown group, suggesting that knockdown PCMT1 may promote the necrosis and apoptosis of tumor tissue. Related studies have shown that PCMT1 may exist in exocrine bodies and vesicles [35, 36], it may have an effect on other cells in the tumor area. Therefore, we further tested the infiltration of CD4+ and CD8+T cells in the tumor area, and found that knockdown of PCMT1 significantly increased the infiltration of CD8+T cells, Activation of CD8+T cells is often considered a key factor in cancer immunotherapy [37], although we did not observe significant changes in CD4+T cells, further subgroup analysis, such as whether there are changes in Treg cells, is also worth further investigation. Besides T cells, relevant literature also showed that macrophages also played an important role in tumor treatment [38–40]. Therefore, we further evaluated the infiltration of M1 and M2 types of macrophages in the tumor area, and the results showed that CD86 expression was significantly increased and CD206 expression was decreased in the tumor area after PCMT1 knockdown, which may indicate that PCMT1 affects the polarization of macrophages in the tumor area, these evidences all suggest that PCMT1 may affect the immune microenvironment of tumors, and inhibition of PCMT1 may better promote anti-tumor immunity (Additional file 3).

## Conclusion

Our study highlights the potential of PCMT1 as a prognostic biomarker and therapeutic target in liver cancer. The modulation of PCMT1 expression appears to influence tumor progression and immune cell infiltration, suggesting a potential synergy between PCMT1-targeting strategies and immunotherapies. Further research is needed to elucidate the precise molecular mechanisms by which PCMT1 affects liver cancer progression and its impact on the immune microenvironment, paving the way for the development of novel therapeutic approaches for liver cancer patients.

### Supplementary Information

**Additional file 1: Figure** **S1** (A-G). The correlation between PCMT1 expression and age, sex, grade and TNM stage.

**Additional file 2: Figure S2**: Association between PCMT1 and tumor mutation burden.

**Additional file 3.** Reagents,antibodies used.

## Data Availability

The data used to support the findings of this study are available from the corresponding author upon request.

## Abbreviations

LIHC: Liver hepatocellular carcinoma
PCMT1: Protein-l-isoaspartate (d-aspartate) *O*-methyltransferase
DEGs: Differentially expressed genes
TMB: Tumor mutation burden
GO: Gene Ontology
KEGG: Kyoto Encyclopedia of Genes and Genomes
