# Supplementary figures and images for: PCMT1 is a potential target related to tumor progression and immune infiltration in liver cancer
Source: Eur J Med Res. 2023 Aug 18;28:289. doi: 10.1186/s40001-023-01216-1 (PMC10436427; doi:10.1186/s40001-023-01216-1)

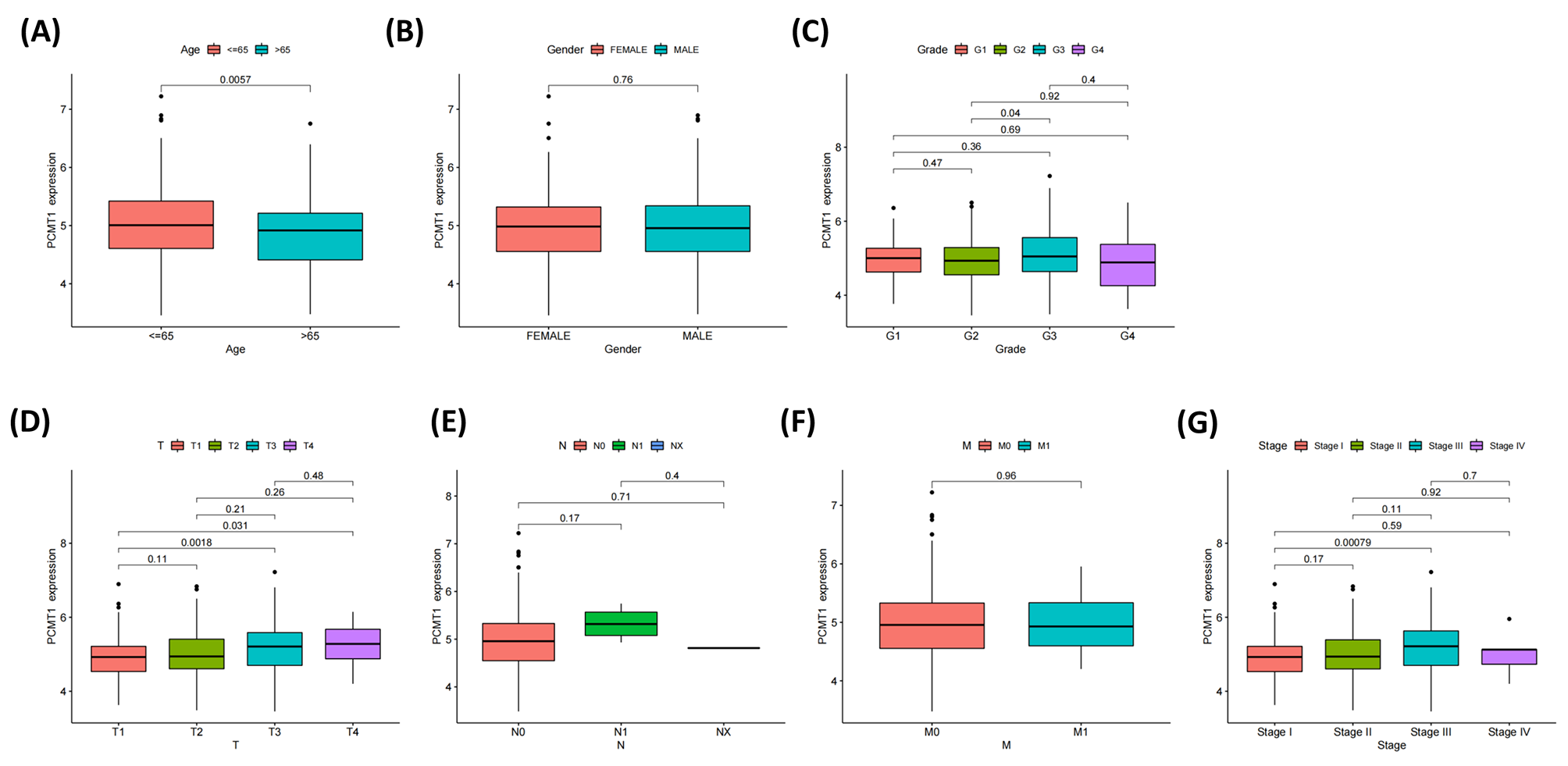

Supplement: Supplementary file 1 — Additional file 1: Figure S1 (A-G). The correlation between PCMT1 expression and age, sex, grade and TNM stage. [file 40001_2023_1216_MOESM1_ESM.tif]

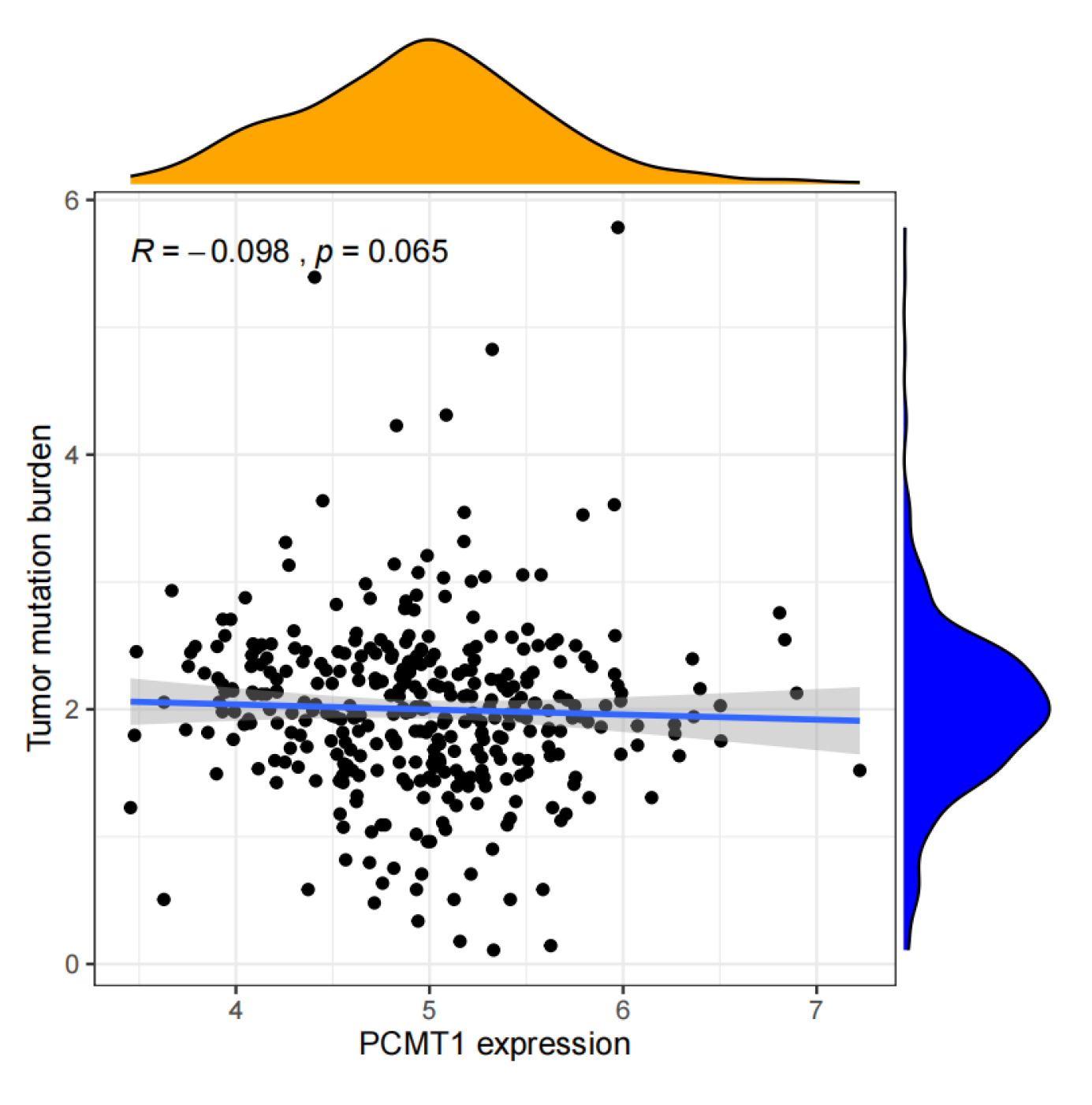

Supplement: Supplementary file 2 — Additional file 2: Figure S2: Association between PCMT1 and tumor mutation burden. [file 40001_2023_1216_MOESM2_ESM.tif]
