# Supplementary material for: PCMT1 is a potential target related to tumor progression and immune infiltration in liver cancer
Source: Eur J Med Res. 2023 Aug 18;28:289. doi: 10.1186/s40001-023-01216-1 (PMC10436427; doi:10.1186/s40001-023-01216-1)
Supplement: Supplementary file 3 — Additional file 3. Reagents,antibodies used. [file 40001_2023_1216_MOESM3_ESM.docx]

**Information about the antibodies used in the experiments**

| Index | Cat No. | COMPANY |
| --- | --- | --- |
| CD45-Alexa700 | 560510 | BD Pharmingen |
| CD4-BV605 | 563151 | BD Pharmingen |
| CD8a-APC-Cy7 | 557654 | BD Pharmingen |
| AnnexinV-FITC/PI | 556547 | BD Pharmingen |
| CD11b | 21851-1-AP | proteintech |
| CD86 | 13395-1-AP | proteintech |
| CD206 | GB113497 | Servicebio |
| Caspase-3 | GB11532 | Servicebio |
| Tunel Apoptosis Detection Kit | G1504 | Servicebio |
| CY3 IgG | GB21303 | Servicebio |
| Alexa Fluor 488 IgG | GB25303 | Servicebio |
| CY5 IgG | GB27303 | Servicebio |
| PCMT1 | 10519-1-AP | proteintech |
| BAX | 50599-2-Ig | proteintech |
| BCL-2 | 26593-1-AP | proteintech |
| p-PI3K | bs-5570R | Bioss |
| p-AKT | ab38449 | abcam |
| E-cadherin | 20874-1-AP | proteintech |
| vimentin | 10366-1-AP | proteintech |
| Goat anti-Rabbit IgG (H+L) Secondary Antibody, HRP | AWS0002 | Abiowell |

**Tumor dissociation kit,mouse:** Miltenyi, order No. 130-096-730
